# Exposure to an Extended-Interval, High-Dose Gentamicin Regimen in the Neonatal Period Is Not Associated With Long-Term Nephrotoxicity

**DOI:** 10.3389/fped.2021.779827

**Published:** 2021-11-30

**Authors:** Veronika Rypdal, Sondre Jørandli, Dagny Hemmingsen, Marit Dahl Solbu, Claus Klingenberg

**Affiliations:** ^1^Department of Pediatrics and Adolescence Medicine, University Hospital of North Norway, Tromsø, Norway; ^2^Pediatric Research Group, Faculty of Health Sciences, University of Tromsø-The Arctic University of Norway, Tromsø, Norway; ^3^The Faculty of Health Sciences, Medical School, University of Tromsø-The Arctic University of Norway, Tromsø, Norway; ^4^Department of Otorhinolaryngology and Head and Neck Surgery, University Hospital of North Norway, Tromsø, Norway; ^5^Section of Nephrology, University Hospital of North Norway, Tromsø, Norway; ^6^Metabolic and Renal Research Group, University of Tromsø-The Arctic University of Norway, Tromsø, Norway

**Keywords:** gentamicin exposure, neonates, subclinical nephrotoxicity, urine biomarkers, chronic kidney disease

## Abstract

**Objectives:** To assess the association between gentamicin exposure and subclinical signs of nephrotoxicity in school children who were exposed to a high-dose gentamicin regimen in the neonatal period.

**Methods:** Children receiving three or more doses (6 mg/kg) of gentamicin as neonates were invited to a follow-up in school age. We evaluated potential signs of subclinical nephrotoxicity with four validated urine biomarkers: protein-creatinine ratio (PCR), albumin-creatinine ratio (ACR), kidney injury molecule-1 (KIM-1), and N-acetyl-beta-D-glucosaminidase (NAG) normalized for urine creatinine (NAG-Cr). In addition, blood pressure was measured. The measures of gentamicin exposure were cumulative dose (mg/kg) and highest trough plasma concentration (TPC) in mg/L. We used logistic and linear regression and non-parametric kernel regression to analyze the relationship between gentamicin exposure and the urine biomarkers.

**Results:** A total of 222 gentamicin exposed children were included. As neonates, the children were exposed to a median (interquartile range-IQR) cumulative gentamicin dose of 36 (26–42) mg/kg and the median (IQR) TPC was 1.0 (0.7–1.3) mg/L. At follow-up, 15 children (6.8%) had either one abnormal urine biomarker value (13 children) or two abnormal urine biomarker values (2 children). These 17 biomarker values were all marginally above the suggested upper cutoff, and included the following markers; KIM-1 (*n* = 2), NAC-Cr (*n* = 5), ACR (*n* = 6), and PCR (*n* = 4). All other 207 children had normal sets of all four urine biomarkers. One child had hypertension. There were no differences in gentamicin exposure, gestational age (GA) at birth or birth weight between the group of 15 children with one or two abnormal urine biomarker values compared to the other 207 children who had normal biomarker values. Using different regression analyses, we did not find any association between gentamicin exposure (cumulative dose and/or TPC) and the urine biomarker values.

**Conclusions:** Exposure to an extended-interval, high-dose gentamicin regimen in the neonatal period was not associated with signs of subclinical nephrotoxicity in schoolchildren. We therefore suggest that the gentamicin treatment regimen evaluated in this study is safe in terms of long-term nephrotoxicity.

**Clinical Trial Registration:** ClinicalTrials.gov, identifier: NCT03253614.

## Introduction

Gentamicin is widely used for treatment of neonatal sepsis (1, 2). To ensure effective therapy with high peak values, it is necessary to attain a high circulating dose. Some experts suggest that each dose should be as high as 7.5 mg/kg due to the large distribution volume in neonates (3), however there is still uncertainty regarding the optimal dosage regimen (4, 5).

There are also conflicting results regarding nephrotoxicity among neonates treated with gentamicin. Several studies report no obvious nephrotoxicity, while others report rates up to 27% (4, 6). The central aspect of gentamicin-induced nephrotoxicity is proximal tubular damage (7). Gentamicin may also in rare cases cause tubulointerstitial fibrosis and mesangial contraction leading to acute kidney injury (AKI) with reduced glomerular filtration rate (GFR) (8).

It has been assumed that nephrotoxicity after gentamicin exposure is reversible, but this has been questioned by studies reporting residual kidney damage after AKI caused by nephrotoxic drugs (9–11). Studies from the last decade report that between 20 and 35% of children beyond the neonatal period exposed to aminoglycosides may develop AKI (12–14). There are also indications of an increased risk of chronic kidney disease (CKD) after AKI among patients in which the kidney function initially seemed completely resolved (9, 10, 15).

In order to identify children at risk for future CKD after gentamicin exposure, urine biomarkers that detect early signs of drug-induced kidney injury, specifically markers of tubulotoxicity, are sought for (16). Children will conserve a normal serum creatinine level until substantial structural damage occurs. Thus, conventional kidney function biomarkers such as serum creatinine and cystatin C may not be suitable for detecting early signs of kidney damage (17, 18).

Protein-creatinine ratio (PCR) and albumin-creatinine ratio (ACR) are widely used urine biomarkers of glomerular injury (19). Kidney injury molecule-1 (KIM-1) and N-acetyl-beta-D-glucosaminidase (NAG) are urine biomarkers of proximal tubular damage (7). Kidney injury molecule-1 seems to be a more specific and sensitive marker for early prediction of AKI than traditionally used biomarkers (20, 21). Studies have also demonstrated that KIM-1 may remain elevated in children after exposure to aminoglycosides, and that higher levels of KIM-1 are associated with higher cumulative aminoglycoside dose (22, 23). Elevated NAG values have also been found in neonates exposed to aminoglycosides (21, 24, 25). Both KIM-1 and NAG are considered to be promising and specific biomarkers for assessment of aminoglycoside-induced tubulotoxicity.

There is sparse long-term data on potential residual kidney damage after gentamicin exposure in the neonatal period. Even less is known about the association between a change in some of the novel urine biomarkers and long-term impacts on kidney function after exposure to nephrotoxic drugs in early childhood. In this study, we assessed possible subclinical signs of nephrotoxicity in schoolchildren who were exposed to a high-dose gentamicin regimen in the neonatal period, in order to assess the long-term safety of this neonatal gentamicin dosage regimen.

## Materials and Methods

### Study Design and Participants

The children included in this study had received gentamicin therapy during their admission in the neonatal intensive-care unit (NICU) at the University Hospital of North Norway, between 2004 and 2012. This NICU is the only unit in the northernmost region of Norway offering care for preterm infants born before 32 weeks of gestation, and other newborns in need of mechanical ventilation or intensive care. We have previously validated and reported data on our extended-interval, high-dose (6 mg/kg) gentamicin dosing regimen (Table 1) (26). We found that 94% of trough plasma concentrations (TPCs) were within the recommended range <2.0 mg/L. Moreover, we found no clear evidence of any clinically relevant early-onset nephrotoxicity when assessing routinely obtained creatinine values, and no signs of early-onset ototoxicity assessed by otoacoustic emission testing (26).

**Table 1:** The extended-interval, high dose neonatal gentamcin dosing regimen evaluated in this study.

**Post-natal** **age (days)**	**Post-menstrual** **age (weeks)**	**Gentamicin dosing** **interval (h)**	**Gentamicin** **dose (mg/kg)**
0–7	<29	48	6
0–7	29–36	36	6
0–7	≥37	24	6
>7	<29	36	6
>7	≥29	24	6

**Figure 1:**
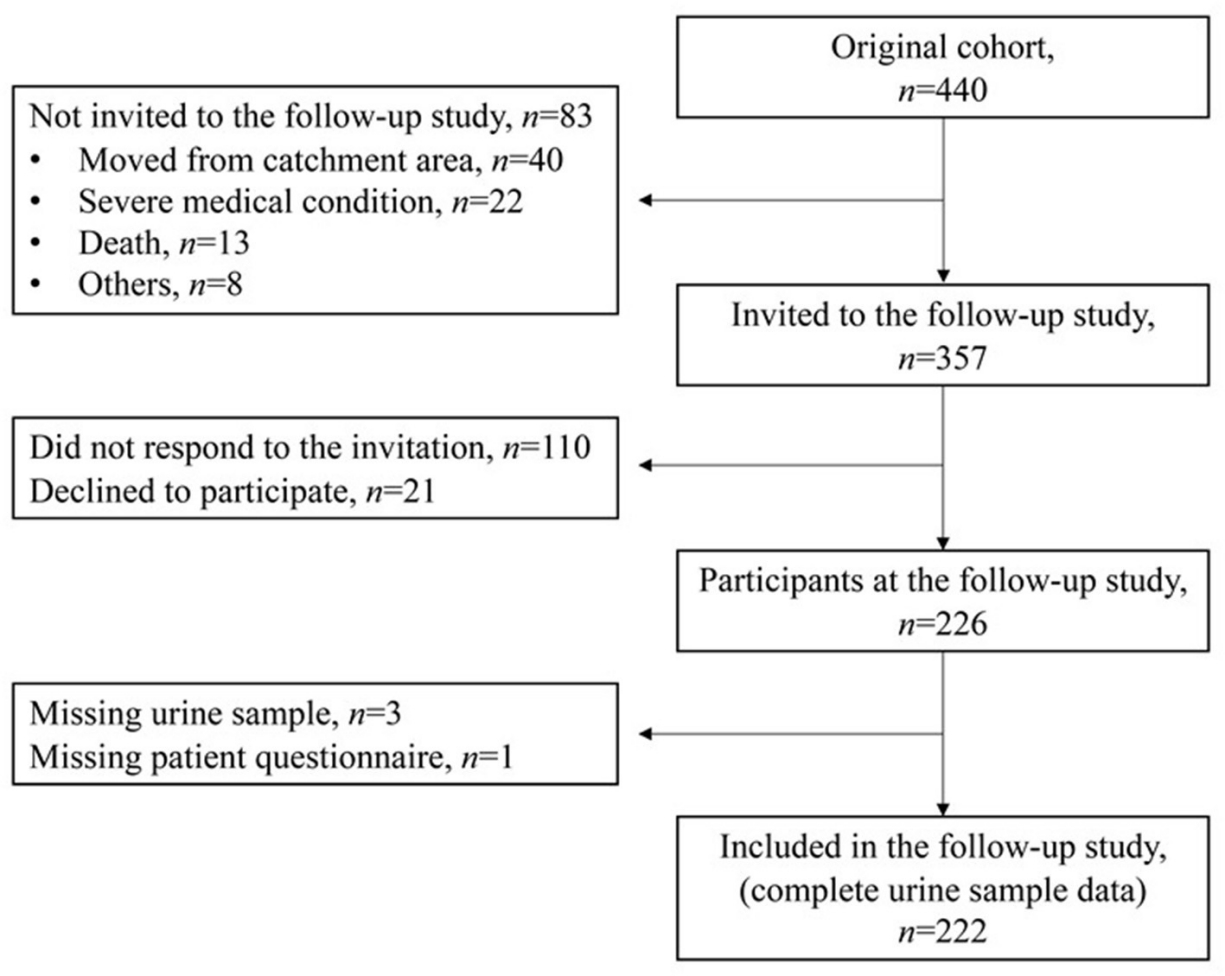
Flow chart of the participants in the study from the original cohort to the present follow-up study.

The original cohort included 440 neonates. For the current study 357 children from the original cohort, still living in North Norway and without major disabilities limiting clinical cooperation, were invited to a follow-up at age of 5–14 years, evaluating possible persistent or late-onset ototoxicity and nephrotoxicity. The follow-up study took place between September 2017 and September 2018, and 226 children came for a 1-day study visit. Data on hearing assessment, including extended high-frequency audiometry, are previously published (27). In this study we present data from the renal evaluation of 222 children. Four children were excluded due to missing urine sample or questionnaire responses (Figure 1).

### Exposure Variables: Gentamicin From the Neonatal Period

We recorded the highest measured gentamicin TPC (mg/L) obtained before the third gentamicin dose, and the cumulative gentamicin dose (mg/kg) administered in the neonatal period.

### Outcome Variables: Urine Biomarkers and Blood Pressure at Follow-Up in School Age

We collected morning spot urine samples and analyzed two urine biomarkers as indicators of tubular dysfunction [KIM-1 and NAG, normalized for urine creatinine (NAG-Cr)], and two urine biomarkers as indicators of glomerular dysfunction (PCR and ACR). KIM-1, PCR, and ACR are recognized by the Predictive Safety Testing Consortium (PSTC), the Food and Drug Agency and the European Medicines Agency as safety urine biomarkers (16, 28). N-acetyl-beta-D-glucosaminidase has not been evaluated by the PSTC, but has been used as a marker of tubular dysfunction or damage in other pediatric studies (29–31).

Kidney injury molecule-1 was analyzed by using the Quantikine^®^ Human Immunoassay (R&D Systems, Inc., Minneapolis, USA), according to the manufacturers' instructions. The limit of quantitation (LOQ) was 0.009 ng/ml. N-acetyl-beta-D-glucosaminidase was analyzed by using a colorimetric assay with 2-methoxy-4-phenyl 2-acetamido-2-deoxy-β-D-glucopyranoside as the substrate (Diazyme Laboratories, CA, USA), according to the manufacturers' instructions. The LOQ was 1.6 U/L. Urine levels of creatinine, protein, and albumin were all analyzed using the Roche/Hitachi cobas c 701/702 platform, which is used in the routine laboratory at the Clinical Chemistry Department at the University Hospital of North Norway. For both PCR and ACR, the laboratory reported exact values if ≥3.0 mg/mmol, and lower values were reported as <3.0 mg/mmol. We decided in our dataset to reclassify all PCR- and ACR-values reported as <3.0 mg/mmol to an exact value of 2.0 mg/mmol.

Pediatric reference values for KIM-1 have been proposed by Bennett et al. (32). KIM-1 values >1.240 ng/ml (age 5–9 years) and >1.141 ng/ml (age 10–14 years) were considered as elevated in our study. Pediatric reference values for NAG-Cr, have been proposed by Muller (33) and Skálová (34). NAG-Cr values >0.7 U/mmol were considered elevated. PCR and ACR value were interpreted in line with the 2012 KIDGO guidelines (19). PCR ≥20 mg/mmol and ACR ≥3.0 mg/mmol were considered elevated.

Blood pressure was measured three times with Philips IntelliVue MP5 bedside patient monitor in a seated position in the right upper extremity with an appropriate cuff size (35). The lowest systolic blood pressure (SBP) and diastolic blood pressure (DBP) were recorded. Hypertension was defined as SBP or DBP >95^th^ percentile for gender and age according to the 2016 European Society of Hypertension guidelines (35).

### Neonatal Characteristics at Baseline and Later Potential Clinical Confounders

From the neonatal period we collected background data on birth weight, gestational age (GA), Apgar scores, and mechanical ventilation in order to correct for severe neonatal disease, as potential confounders that could have an impact on renal function. In order to assess other potential confounders after the neonatal period, we recorded from a parental questionnaire data on antibiotic therapy after the neonatal period, the occurrence of urinary tract infections (UTI), reported kidney disease and family history of kidney disease. To assess representativeness of the follow-up cohort regarding gentamicin exposure and other clinical variables, we compared data from the original cohort (*n* = 440; neonates) with data from the follow-up cohort (*n* = 222; now school children).

### Sample Size and Power Calculation

Sample size and power calculation were estimated for the ototoxicity part of this follow-up study (27). No separate sample size estimate for assessment of subclinical nephrotoxicity was performed.

### Data Analysis and Statistics

All clinical data were first entered into REDCap^®^ (Vanderbilt University, Nashville, USA). Statistical analyses were performed with Stata/MP version 16. We used medians and interquartile ranges (IQRs) to describe demographics and clinical characteristics. Differences between groups were analyzed using the chi-square test or Fisher's exact test, where appropriate, for dichotomized variables, and the Mann-Whitney U-test for continuous variables. Univariate logistic regression analysis with odds ratios (OR) was used to assess gentamicin exposure (cumulative dose and/or TPC) and other clinical variables (confounders) as possible risk factors of subclinical nephrotoxicity. Both linear regression and non-parametric kernel regression were used to analyze the relationship between gentamicin exposure and the different urine biomarker values. Non-parametric regression does not assume a linear relationship between predictors (gentamicin exposure) and outcome (subclinical nephrotoxicity) and may therefore discern associations in the data that are not detected using linear regression. In the non-parametric regression, we used Epanechnikov kernel functions and the association between urine biomarkers and gentamicin exposure was quantified as the average derivative of the estimated curve. The significance was estimated by bootstrapping. The goodness-of-fit measure for the regression models was presented with *R*^2^.

Since all PCR- and ACR-values <3 mg/mmol were reclassified to the exact value 2 mg/mmol, this resulted in a clustering of the lowest values for these two outcome variables. Since the distributions of gentamicin exposure and urine biomarkers had high kurtosis, we decided to present the data using double-logarithmic plots, i.e., with logarithmic scales on both the x- and the y-axes.

### Ethics Approval and Trial Registration

The study was approved by the Regional Committee for Medical and Health Research Ethics, Region North in Norway. Participating children received age-appropriate written information about the study and all the parents signed a written informed consent to participate in the study, including consent for the publication of data without any personal identification of participants. The study was conducted according to the guidelines of the Declaration of Helsinki.

The trial was registered at ClinicalTrials.gov, NCT03253614.

## Results

### Characteristics of Included Participants

A total of 222/357 (62%) of the gentamicin-exposed and invited children were included (Figure 1). The majority (143/222; 64%) were term born (≥37 weeks gestation). Among the 79 (36%) participants that were born prematurely, 48 were born <32 weeks gestation, 41 had a birth weight <1,500 g, and 21 were defined as being small for GA (<10^th^ percentile) at birth. A total of 48/222 (22%) children had received mechanical ventilation in the neonatal period, as an indicator of more severe neonatal disease. When we compared the original cohort of neonates to the children included in the follow-up study there were no significant differences in birth weight, the proportion of infants with a birth weight <1,500 g, the cumulative gentamicin doses, the highest median gentamicin TPCs and the proportion of children with gentamicin TPC >2.0 mg/L between the two cohorts (Table 2).

**Table 2:** Comparison of clinical characteristics and gentamicin exposure in the original cohort and the follow-up cohort.

**Clinical characteristics**	**Original cohort**	**Follow-up cohort**
	***n*** **= 440**	***n*** **= 222**
Gestational age, weeks	39 (32–40)	39 (33–40)
Birth weight, g	3,281 (1,850–3,815)	3,337 (1,996–3,890)
Birth weight <1,500 g, *n* (%)	84 (19.0)	41 (18.5)
Gentamicin TPC, mg/L	1.0 (0.7–1.3)	1.0 (0.7–1.3)
TPC ≤ 1 mg/L, *n* (%)	252 (57.3)	129 (58.1)
TPC >1.0 mg/L, *n* (%)	188 (42.7)	93 (41.9)
TPC >2.0 mg/L, *n* (%)	26 (6.0)	12 (5.4)
Gentamicin cumulative dose, mg/kg	30 (24–36)	36 (24–42)
Age at follow-up, years		9 (7–11)
Body mass index at follow-up, kg/m^2^		17.4 (15.8–20.4)

*Values are presented as median with interquartile range (IQR) if not otherwise specified. TPC, trough plasma concentration. There were no significant differences in any of the presented variables between the original cohort and the follow-up cohort*.

At the follow-up visit the median age was 9 years and there were more boys (*n* = 137) than girls (*n* = 85). Parents reported that seven children had a “kidney disease in the family” and that one child had a current “kidney disease,” but none of these had any abnormal urine biomarkers. Fourteen children had recurrent UTI; one of them had an abnormal KIM-1 value, but all other urine biomarkers were normal in this child. After the neonatal period 32 children had been treated with some kind of antibiotics (not specified). Among these, three children had abnormal urine biomarkers: one elevated PCR and ACR, one elevated ACR only, and one elevated KIM-1 only. All abnormal markers in these children were values marginally above the suggested cutoffs.

### Gentamicin Exposure, Urine Biomarkers, and Blood Pressure

Data on gentamicin exposure are presented in Table 1. The median (IQR) cumulative gentamicin dose in the neonatal period was 36 (24–42) mg/kg, which reflects a treatment course of 4–7 gentamicin doses for suspected or confirmed neonatal sepsis.

Twelve children had received a very high cumulative gentamicin dose (>78 mg/kg) as neonates, which equals 13 or more gentamicin doses. Among these 12 children, the median (IQR) GA was 26 (25–30) weeks and the median (IQR) birth weight was 902 (764–1,035) g. Eight of these children also received mechanical ventilation. One child among these 12 had a single abnormal urine biomarker: a NAG-Cr value of 1.05 U/mmol (Table 2). This particular child had received a cumulative gentamicin dose of 366 mg/kg, but all other urine biomarkers, and the blood pressure, were normal in this child.

Twelve children had an elevated gentamicin TPC (>2.0 mg/L) during the treatment course in the neonatal period. One child among these 12 had an isolated mildly elevated ACR (4.0 mg/mmol).

In Table 3 we present clinical characteristics, urine biomarker levels, and blood pressure data from the 15 children (6.8%) who had at least one elevated urine biomarker. Two children had mildly elevated values for both PCR and ACR. The other 13 children had a single elevated biomarker, with all other urine biomarkers below the suggested cutoff. We compared the 15 children with at least one elevated urine biomarker with the 207 children who had normal values for all four urine biomarkers. There were no significant differences in the median GA, birth weight, age at follow-up, the rates of UTI, proportion of children receiving cumulative gentamicin doses ≥36 mg/kg or proportion of children who had gentamicin TPC >2.0 mg/L. Ten of the 15 children with at least one elevated urine biomarker were girls. In the total cohort, two children (1%) had SBP >95^th^ percentile, and one of these also had DBP >95^th^ percentile for age and gender (35). Among the 48 children born <32 weeks GA, two had one single, isolated elevated urine biomarker each, and one of them also had hypertension (Table 3). Overall, we analyzed 888 urine biomarkers from the 222 children, and only 17 of 888 tests came out with a value above cutoff.

**Table 3:** Characteristics of the 15 children with one (*n* = 13) or two (*n* = 2) abnormal urine biomarkers at the follow-up visit.

**Gender**	**GA** **(w)**	**BW** **(g)**	**Mechanical** **ventilation**	**Antibiotics^a^**	**Gentamicin** **cumulative dose** **(mg/kg)**	**Gentamicin** **TPC** **(mg/L)**	**KIM-1** **(ng/ml)**	**NAG-Cr** **(U/mmol)**	**ACR** **(mg/mmol)**	**PCR** **(mg/mmol)**	**Hypertension^b^**
♀	40	3,408	No	Yes	18	1.9	1.27^*^	0.20	2	12	No
♀	39	3,960	No	No	36	1.0	0.15	0.74^*^	2	2	No
♀	35	2,154	No	No	30	0.5	2.15^*^	0.24	2	11	No
♂	40	4,262	No	No	36	0.8	0.15	0.84^*^	2	2	No
♀	40	4,300	No	No	36	0.9	0.15	0.22	8^*^	20^*^	No
♂	40	2,940	No	No	30	1.0	0.40	0.18	3^*^	17	No
♀	39	3,790	No	No	36	2.6	0.16	0.12	4^*^	15	No
♀	39	3,315	No	Yes	42	0.8	0.15	0.18	9^*^	24^*^	No
♂	39	3,580	No	No	36	1.1	0.15	0.17	4^*^	15	No
♀	38	3,315	No	No	24	0.7	0.15	0.17	2	20^*^	No
♂	40	4,184	No	No	30	0.7	0.15	0.16	3^*^	16	No
♀	26	850	Yes	No	366	1.0	0.15	1.05^*^	2	2	No
♂	39	3,350	No	No	66	0.7	0.15	0.80^*^	2	2	No
♀	30	1,455	Yes	Yes	48	0.5	0.15	0.19	2	20^*^	Yes
♀	39	2,780	No	No	24	1.8	0.15	0.82^*^	2	15	No

*TPC, trough plasma concentration; KIM-1, Kidney injury molecule-1; NAG/Cr ratio, N-acetyl-β-D-glucosaminidase (NAG) creatinine ratio; ACR, albumin to creatinine ratio; PCR, protein to creatinine ratio; GA, gestational age (weeks); BW, birth weight (g).*

* *Abnormal value of the urine biomarker. Cutoffs used in this study: KIM-1 >1.240 ng/ml (age 5–9 years) and >1.141 ng/ml (age 10–14 years), NAG-Cr >0.7 U/mmol, ACR ≥3.0 mg/mmol, and PCR ≥20 mg/mmol.*

a *Antibiotics after the neonatal period.*

b *Systolic or diastolic blood pressure >95^th^ percentile*.

### Regression Analyses Assessing Level of Gentamicin Exposure and Urine Biomarkers

We did not find any association between gentamicin exposure, nor other possible risk factors of nephrotoxicity, and the level of the urine biomarkers PCR, ACR, and KIM-1 at the follow-up visit in univariate logistic regression analysis (Table 4). For NAG-Cr there was an association between the cumulative dose gentamicin and NAC-Cr level (OR 1.18, 95% CI 1.04–1.34, *p* = 0.01), but this was solely due to one child who received a cumulative dose of 366 mg/kg, i.e., an outlier in the dataset. After removing this outlier, the association was no longer present (OR 1.03, 95% CI 0.71–1.50, *p* = 0.86).

**Table 4:** Univariate logistic regression of gentamicin exposure and other baseline characteristics assessed for the associated with abnormal urine biomarkers.

	**ACR**		**PCR**		**KIM-1**		**NAG-Cr**	
	**(mg/mmol)**		**(mg/mmol)**		**(ng/ml)**		**(U/mmol)**	
**Baseline characteristics**	**OR (95%CI)**	***p***	**OR (95% CI)**	***p***	**OR (95% CI)**	***p***	**OR (95% CI)**	***p***
Gentamicin, mg/kg^a^	0.92 (0.60–1.41)	0.70	0.97 (0.65–1.44)	0.88	0.36 (0.06–2.24)	0.27	1.18 (1.04–1.34)	0.01^b^
Gentamicin, TPC^c^	1.37 (0.40–4.70)	0.62	0.18 (0.01–2.73)	0.22	1.41 (0.18–11.2)	0.74	0.98 (0.21–4.61)	0.98
Gender	1.63 (0.32–8.29)	0.55	NA	–	NA		2.47 (0.40–15.1)	0.33
Gestational age, weeks	1.21 (0.90–1.63)	0.21	1.01 (0.83–1.22)	0.93	1.04 (0.77–1.40)	0.79	1.00 (0.85–1.19)	0.98
Birth weight, g^d^	1.10 (0.98–1.16)	0.15	1.01 (0.93–1.10)	0.84	0.98 (0.88–1.10)	0.82	1.00 (0.93–1.08)	0.91
Small for gestational age	0.51 (0.06–4.61)	0.55	NA	–	0.10 (0.01–1.67)	0.11	NA	–
Mechanical ventilation	NA		0.82 (0.08–8.11)	0.87	NA		1.11 (0.12–10.1)	0.93

*ACR, albumin to creatinine ratio (mg/mmol); PCR, proteine to creatinine ratio (mg/mmol); KIM-1, kidney injury molecule-1 (ng/ml); NAG-Cr, N-acetyl-β-D-glucosaminidase (NAG) creatinine ratio (U/mmol); OR, odds ratio; CI, confidence interval; NA, not applicable.*

a *Gentamicin cumulative dose (mg/kg) per 10 mg/kg increase in dose.*

b *After removing one patient with cumulative gentamicin dose of 366 mg/kg there were no significant association between cumulative gentamicin and NAG-Cr ratio (OR 1.03; 95% CI 0.71–1.50, p = 0.86).*

c *TPC (trough plasma concentration) in mg/L.*

d *Birth weight in gram (g) per 100 g increase in weight*.

We also performed linear regression analyses to evaluate any associations between gentamicin exposure and urine biomarkers. The *R*^2^-values were <0.001 for KIM-1, PCR, and ACR and 0.004 for NAG-Cr. The *p*-values for the coefficient in the linear regressions were 0.73 for KIM-1, 0.80 for PCR, 0.89 for ACR, and 0.31 for NAG-Cr.

**Figure 2:**
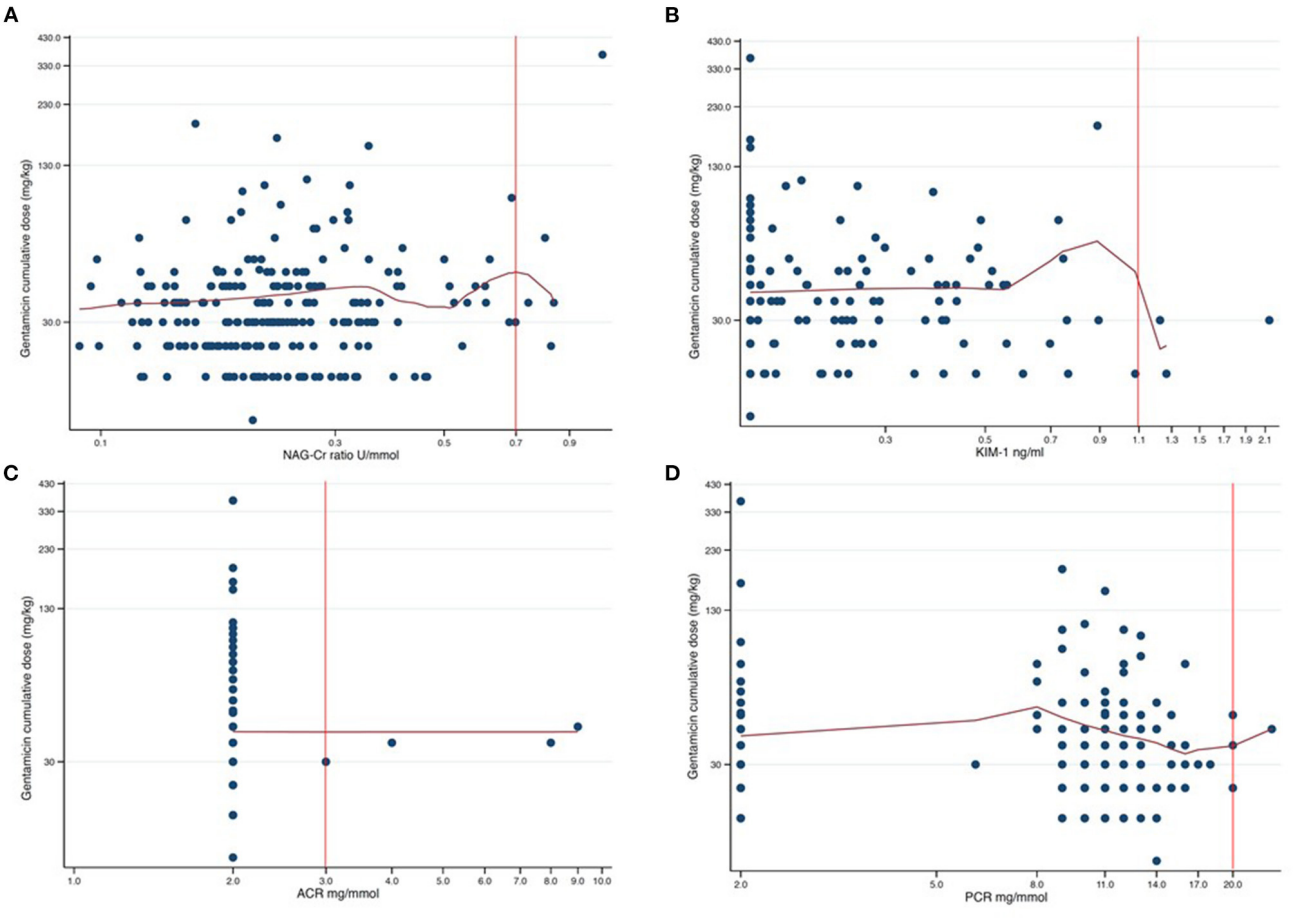
Scatter plots of cumulative dose gentamicin against each of the four assessed urine biomarkers presented in a log-log graph. The red curves are the results from non-parametric kernel regression for the relationship between the cumulative dose of gentamicin and the urine biomarkers. The y-axis shows the cumulative dose of gentamicin in mg/kg with 100 mg/kg between axis ticks in each of the plots. The red vertical lines are the respective cutoff values for abnormal urine biomarker levels. The tick markers for the x-axes are evenly spaced but appear unevenly spaced since the axes are logarithmic. The distance between axes ticks is chosen so that, in each plot, one tick marker coincides with the cutoff value (red vertical line). **(A)** NAG-Cr (U/mmol) on a logarithm scale, with the distance between the tick markers for the x-axis of 0.2 U/mmol. **(B)** KIM-1 (ng/ml) on a logarithm scale, with the distance between the tick markers for the x-axis of 0.2 ng/ml. **(C)** ACR (mg/mmol) on a logarithm scale, with the distance between the tick markers for the x-axis of 1.0 mg/mmol. **(D)** PCR (mg/mmol) on a logarithm scale, with the distance between the tick markers for the x-axis of 3.0 mg/mmol.

**Figure 3:**
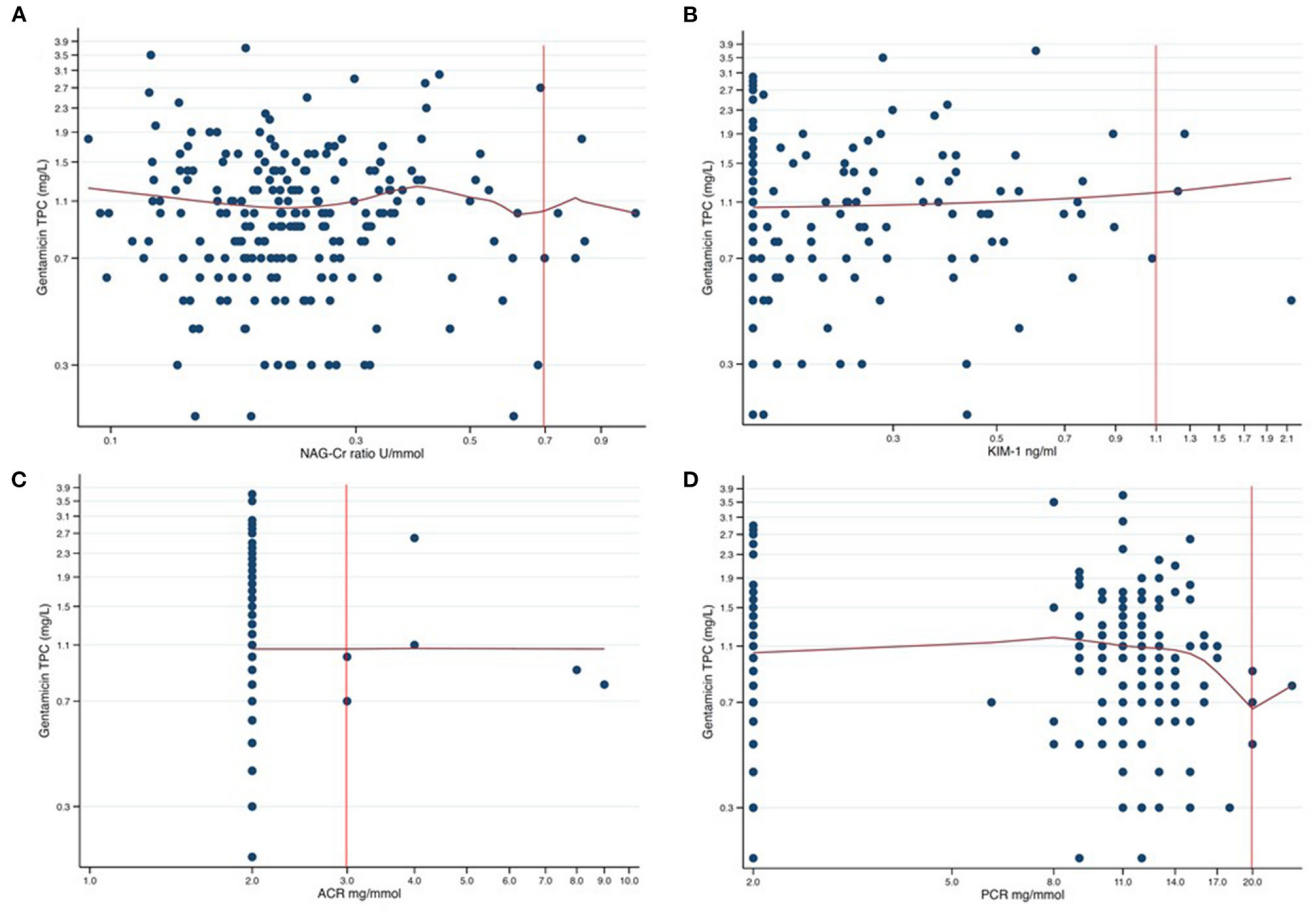
Scatter plots of gentamicin trough plasma concentration (TPC) against each of the four assessed urine biomarkers presented in a log-log graph. The red curves are the results from non-parametric kernel regression for the relationship between the gentamicin TPC and the urine biomarkers. The y-axis shows the gentamicin TPC in mg/L with 0.4 mg/L between axis ticks in each of the plots. The tick markers for the x-axes are evenly spaced but appear unevenly spaced since the axes are logarithmic. The distance between axes ticks is chosen so that, in each plot, one tick marker coincides with the cut-off value (red vertical line). **(A)** NAG-Cr (U/mmol) on a logarithm scale, with the distance between the tick markers for the x-axis of 0.2 U/mmol. **(B)** KIM-1 (ng/ml) on a logarithm scale, with the distance between the tick markers for the x-axis of 0.2 ng/ml. **(C)** ACR (mg/mmol) on a logarithm scale, with the distance between the tick markers for the x-axis of 1.0 mg/mmol. **(D)** PCR (mg/mmol) on a logarithm scale, with the distance between the tick markers for the x-axis of 3.0 mg/mmol.

We finally performed non-parametric kernel regressions analyses to further evaluate any possible associations between gentamicin exposure and the four urine biomarkers (Figures 2, 3). We found no associations between gentamicin exposure and the level of the four urine biomarkers. The *R*^2^-values were 0.01, 0.02, <0.01, and 0.01 for NAG-Cr, KIM-1, ACR, and PCR, respectively. The *p*-values were >0.05 for all possible associations.

## Discussion

The main objective of this study was to perform a detailed assessment of urine biomarkers in schoolchildren exposed to an extended-interval, high-dose gentamicin regimen during the neonatal period in order to assess potential subclinical signs of nephrotoxicity as markers of long-term harm or safety. We used established urine biomarkers as indicators for both glomerular and tubular damage and adjusted our findings for other potential peri- and post-natal risk factors for kidney damage. The vast majority of schoolchildren in this study had normal urine biomarkers and blood pressure, and we found no association between level of gentamicin exposure in the neonatal period and subclinical nephrotoxicity in school-age.

Aminoglycosides, such as gentamicin, are particularly active against Gram-negative bacteria (12) and have an important role in neonatal sepsis regimens (36). Narrow-spectrum regimens, containing aminoglycosides and penicillin or ampicillin, are associated with lower risk of antimicrobial resistance (AMR) development in the neonatal period compared to broad-spectrum antibiotics (37). Antimicrobial resistance is a global public health threat and overuse of antimicrobials are the main drivers of AMR-development (38). Carbapenem-regimens may offer a better coverage than ampicillin and gentamicin in some low- and middle-income countries (39), but their use are accompanied with the vicious cycle of further increasing AMR. Aminoglycoside still offer a reasonably good coverage of Gram-negative bacteria (40) and further optimizing neonatal gentamicin dosing regimens and considering the use of other members of the aminoglycoside-family should be considered before moving to antibiotic-classes driving AMR (41, 42). We therefore believe that long-term safety data may support clinical decision-making in favor of effective and safe aminoglycoside-based regimens.

In line with some others (43), we found no signs of chronic tubular dysfunction after prior therapy with aminoglycosides. In contrast, in a retrospective study of children who developed nephrotoxin-induced AKI (after ≥3 days of aminoglycosides or ≥3 nephrotoxins simultaneously for 1 day), the relative risk of developing signs of CKD 6 months later was 3.84 (95% CI 1.57–9.40) compared to nephrotoxin-exposed controls who did not develop AKI (9). However, AKI after gentamicin exposure in neonates is probably rare and we did not diagnose AKI in the neonatal period in our cohort. There may also be other reasons for the favorable and reassuring findings in our study. Even though median cumulative dose in our study was 36 mg/kg, this reflects only six doses administered over 6 days, or longer in the preterm infants. In most studies there has been an association between prolonged aminoglycoside use, often longer than in our study, and nephrotoxicity (12, 14). However, in our regression analyses we did not find any signs of a dose-response nephrotoxicity neither with increasing cumulative doses nor with higher TPCs. Extended-interval dosing regimens are also probably safer that multiple daily dosing in terms of risk of acute nephrotoxicity (44, 45).

In our follow-up cohort only 79 preterm infants were included, of which 48 were born before 32 weeks of gestation. Nephrogenesis continues until about 34–36 weeks of gestation with more than 60% of nephrons being formed in the last trimester of pregnancy (46). Preterm infants are at increased risk of neonatal AKI as well as higher blood pressure, proteinuria, and CKD later in life (47). To our surprise we did not see any link between lower GA and signs of tubular or glomerular damage in our cohort. Aminoglycosides exert their nephrotoxicity within the proximal tubule epithelial cells (12). It is possible that immature proximal tubules may be less prone to megalin-induced accumulation of aminoglycosides and thus to some extent protect the preterm infants from gentamicin-induced nephrotoxicity.

The strengths of our study are the unique long-term follow-up with broad evaluation of subclinical nephrotoxicity, using safety urine biomarkers and blood pressure measurement. Paradoxically, most neonatal gentamicin dosing regimens recommend lower gentamicin doses (4–5 mg/kg) than in older children (7–8 mg/kg), despite a proportionally higher volume of distribution in neonates (48). Since 2004 we have used a dosing regimen in our NICU with a fixed gentamicin dose of 6 mg/kg for all neonates, and a variable dosing interval (24–48 h) depending on GA and post-natal age (26). With this dosing regimen we have not found any evidence of long-term ototoxicity (26, 27), and the present study also suggest that this dosing regimen is associated with long-term renal safety and no signs of long-term nephrotoxicity. There is limited previous data on biomarkers of CKD in the pediatric population, and our data presenting urine biomarkers capturing different aspects of potential renal damage after aminoglycoside therapy in the neonatal period are novel.

Our study also has limitations. The most important limitation is a response rate of 63% which adds a potential selection bias. However, there were no significant differences in gentamicin exposure nor the proportion of very low birth weight infants when comparing children attending the follow-up study to those not included in the follow-up, implying that these patients probably were similar. Second, we did not collect urine samples for biomarker analysis from a healthy control group. We therefore had to rely on comparison with previous reported reference values and cutoffs for the urine biomarkers. For KIM-1, ACR, and PCR, we believe the proposed cutoffs are based on solid clinical evidence. Indeed, some studies have reported slightly higher, upper cutoffs for KIM-1 according to the 95^th^ percentile for age (30, 49). Reported cutoffs for NAG-Cr ratio varied more in the literature and are clearly age-dependent with the highest values in neonates and infants (29, 33). However, in the age group 5–14 years from our study, reported cutoffs are relatively stable. Thus, we believe our suggested NAG-Cr ratio cutoff at 0.7 U/mmol is reasonable, but it still remains unclear whether NAG should be normalized for creatinine or not (30). Recent studies report that the predictive value of KIM-1 is better than NAG, and the very low rate of abnormal KIM-1 values in our study supports our conclusion regarding no signs of long-term tubular damage among children exposed to gentamicin as neonates. Third, we did not measure serum creatinine and thus could not estimate GFR at the follow-up visit. The reason for omitting blood tests from the protocol was the possibility of parents refusing to let their child participate in the follow-up due to painful procedures. This could have led to a less representative sample size. Moreover, we investigated subclinical nephrotoxicity, and elevation of creatinine several years after gentamicin exposure is unlikely. Fourth, nephrotoxicity is particularly challenging to interpret in the neonatal period when plasma creatinine values are influenced by maternal values, renal maturity, and changes in the systemic circulation of sick neonates (50). In our original study (26) we did not identify neonates with “clinically relevant” gentamicin-induced AKI but we did not use a standardized definition for neonatal AKI, with measurement of urine output and serial creatinine measurements. Fifth, only 10% of the children in our study received more than 10 doses (>60 mg/kg) gentamicin, and we cannot exclude that very long courses of gentamicin have a greater nephrotoxic potential, also in the neonatal period. Sixth, we should ideally have repeated the urine samples in the children with biomarker values above the defined cutoffs. Studies have e.g., shown that proteinuria in a spot urine sample may randomly be found in 10% of school-age children, due to orthostatic or transient proteinuria. By repeating the urine sample the rate of positive tests may be reduced considerably (51, 52). In our study, only 8 of 222 children had proteinuria, all of mild degrees, and we cannot rule out that these samples had been negative if repeated. Nevertheless, this would not change the conclusions of this study.

## Conclusion

We found no association between neonatal exposure to a gentamicin high-dose, extended-interval gentamicin dosing regimen, and increased risk of later subclinical nephrotoxicity in school-age, using urine biomarkers assessing tubular and glomerular dysfunction and blood pressure measurements as a possible sign of renal hypertension.

## Data Availability Statement

The raw data supporting the conclusions of this article will be made available by the authors, without undue reservation.

## Ethics Statement

The studies involving human participants were reviewed and approved by Regional Committee for Medical and Health Research Ethics, Region North in Norway. Written informed consent to participate in this study was provided by the participants' legal guardian/next of kin.

## Author Contributions

VR performed the statistical analysis and wrote the first draft of the manuscript. SJ participated in data curation and analyses, screened the literature, and reviewed the manuscript for intellectual content. DH participated in design of the study, coordinated and supervised data collection, and reviewed the manuscript for intellectual content. MS reviewed the manuscript for intellectual content and revised the manuscript. CK conceptualized and designed the study, coordinated and supervised data collection, directed all phases of the study, and revised the final manuscript. VR and CK had full access to all of the data in the study and takes responsibility for the integrity of the data and the accuracy of the data analysis. All authors approved the final manuscript as submitted and agree to be accountable for all aspects of the work.

## Funding

This study was funded by Northern Norway Regional Health Authority and by the Research Department at University Hospital of North-Norway.

## Conflict of Interest

The authors declare that the research was conducted in the absence of any commercial or financial relationships that could be construed as a potential conflict of interest.

## Publisher's Note

All claims expressed in this article are solely those of the authors and do not necessarily represent those of their affiliated organizations, or those of the publisher, the editors and the reviewers. Any product that may be evaluated in this article, or claim that may be made by its manufacturer, is not guaranteed or endorsed by the publisher.

## Abbreviations

ACR: albumin-creatinine ratio
AKI: acute kidney injury
CKD: chronic kidney disease
GA: gestational age
GFR: glomerular filtration rate
NAG: N-acetyl-beta-D-glucosaminidase
KIM-1: kidney injury molecule-1
PCR: protein-creatinine ratio.
